# Feasibility, effectiveness, and safety of edoxaban administration through percutaneous endoscopic gastrostomy: 12-months follow up of the ORIGAMI study

**DOI:** 10.3389/fcvm.2022.1052053

**Published:** 2022-12-22

**Authors:** Luigi Cappannoli, Renzo Laborante, Mattia Galli, Francesco Canonico, Giuseppe Ciliberti, Attilio Restivo, Giuseppe Princi, Alessandra Arcudi, Mario Sabatelli, Raimondo De Cristofaro, Filippo Crea, Domenico D’Amario

**Affiliations:** ^1^Dipartimento di Scienze Cardiovascolari, Fondazione Policlinico Universitario A. Gemelli IRCCS, Rome, Italy; ^2^Università Cattolica del Sacro Cuore (UCSC), Rome, Italy; ^3^Gruppo Villa Maria (GVM) Care & Research, Maria Cecilia Hospital, Cotignola, Italy; ^4^Centro NEuroMuscular Omnicenter (NEMO), Fondazione Policlinico Universitario A. Gemelli IRCCS, Rome, Italy; ^5^Servizio Malattie Emorragiche e Trombotiche, Dipartimento di Medicina e Chirurgia Traslazionale, Fondazione Policlinico Universitario A. Gemelli IRCCS, Rome, Italy

**Keywords:** edoxaban, percutaneous endoscopic gastrostomy, atrial fibrillation, oral anticoagulant, bleeding, fragile patients

## Abstract

**Background and aims:**

Edoxaban proved to be safe and effective also in fragile patients, but its administration through percutaneous endoscopic gastrostomy (PEG) has not been previously investigated. The purpose of this study was to evaluate the feasibility and the preliminary safety and efficacy profiles of edoxaban administered *via* PEG in patients with an indication for long-term oral anticoagulation.

**Methods:**

ORIGAMI was a prospective, single-arm, observational study (NCT04271293). Patients with PEG and an indication for long-term anticoagulation were prospectively enrolled. Crushed edoxaban at approved doses was administered *via* PEG. The primary endpoint was the composite of cardio-embolic events consisting of ischemic stroke, systemic embolism, or symptomatic deep venous thrombosis/pulmonary embolism (DVT/PE). Secondary endpoints were the number of bleeding events and edoxaban plasma concentrations at steady state. We here report the 12-month results.

**Results:**

A total of 12 patients were enrolled. The main indication for PEG implantation was amyotrophic lateral sclerosis (10/12). The primary endpoint of cardio-embolic events did not occur in any patients at 12 months. All patients were in the therapeutic range of steady-state edoxaban plasma levels. Three minor bleedings were observed, while no major bleedings occurred during the observational period. A total of five patients died. All deaths were from non-cardiovascular causes and were consistent with the natural history of the pre-existing severe disease.

**Conclusion:**

Our study suggests that edoxaban administration *via* PEG is feasible and appears safe and effective in fragile, comorbid patients, resulting in therapeutic plasma concentrations of edoxaban.

**Clinical trial registration:**

[ClinicalTrials.gov], identifier [NCT04271293].

## Introduction

Direct oral anticoagulants (DOACs) are recommended by current guidelines over vitamin K antagonists (VKA) for stroke prevention in patients with non-valvular atrial fibrillation (NVAF) and for treatment and prevention of deep venous thrombosis/pulmonary embolism (DVT/PE) (1, 2).

The use of oral anticoagulants in fragile (i.e., elderly, low body weight, cancer, severe comorbid) patients is a challenge for clinicians due to the need to accurately balance the risk-benefit ratio between thromboembolic risk and bleeding complications. Among the four available DOACs, edoxaban has already proved to be effective and safe in patients with active malignancies, in very elderly patients, and in those suffering from frailty in general (3–5). Recently, these results have been confirmed by a sub-analysis of the ELDERCARE-AF randomized trial in fragile patients and by a real-world retrospective study in cancer patients (6, 7). Whether edoxaban may be effectively and safely administered through percutaneous endoscopic gastrostomy (PEG) in complex patients is, however still to be proved.

Percutaneous endoscopic gastrostomy is a long-term feeding option for patients with the inability to swallow or with upper digestive tract obstruction, such as those with oncological or neurological disease (8). For these patients, administration of oral treatments is made through PEG as well, usually in a crushed form. Despite current guidelines reporting that the administration of edoxaban in crushed form by nasogastric tube does not modify its bioavailability, it is not mentioned whether it may be safely administered *via* PEG (1). Considering that an increasing number of chronic conditions may lead fragile patients to PEG implantation and that they often present a concomitant indication for long-term anticoagulation (mainly atrial fibrillation, but also DVT/PE), data in support of the fact that edoxaban can be safely administered through this route represents an urgent unmet need.

Based on our previous experiences with the administration of crushed edoxaban through PEG in fragile patients with amyotrophic lateral sclerosis (ALS), we designed the ORIGAMI pilot study to evaluate the feasibility, and preliminary safety and efficacy profile of edoxaban administered *via* PEG in patients with an indication for long-term oral anticoagulation (9, 10).

## Materials and methods

**FIGURE 1:**
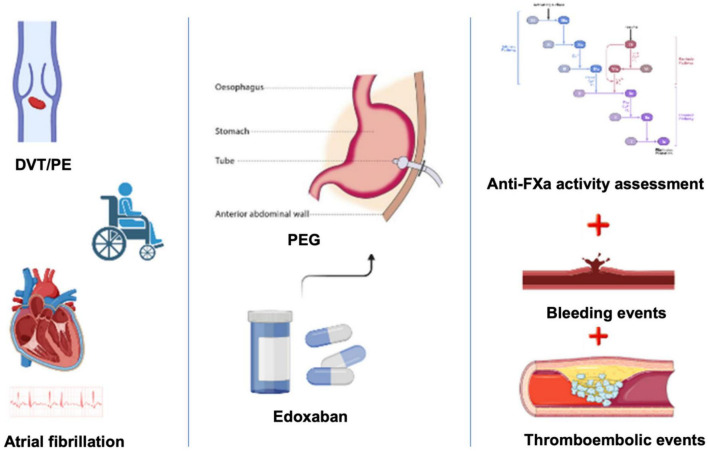
The main features of the ORIGAMI study design. Patients with indication for long-term oral anticoagulation indication were enrolled; edoxaban (at approved dose) was administered through PEG; thromboembolic and bleeding events and anti-FXa activity were assessed at 1, 6, and 12 months. DVT/PE, deep vein thrombosis/pulmonary embolism; PEG, percutaneous endoscopic gastrostomy.

The materials and methods of ORIGAMI (ORal anti-coagulants In fraGile patients with percutAneous endoscopic gastrostoMy and atrIal fibrillation) pilot study were previously described (10, 11). Briefly, ORIGAMI was a prospective, single-arm, observational, pilot study. ClinicalTrials.gov identifier: NCT04271293, and it has been approved by the local ethical committee of Fondazione Policlinico Universitario Agostino Gemelli IRCCS (protocol number 49,640/19, ID 2,897). Consecutive patients with PEG and guideline-recommended indication for long-term anticoagulation were prospectively enrolled. Inclusion and exclusion criteria are listed in Table 1. Crushed edoxaban, diluted in 10 ml of saline solution, was administered with a syringe *via* PEG once daily (od). A dose adjustment to 30 mg/od was made in case of eGFR < 50 ml/min, a body weight of 60 kg or less, and in case of concomitant use of P-glycoprotein inhibitors. Drug steady-state concentration was measured by collecting peripheral blood samples 2–4 h after the administration of at least the fourth dose of edoxaban and measuring the anti-Xa activity. The primary endpoint was a composite of cardio-embolic events consisting of ischemic stroke, systemic embolism, or symptomatic DVT/PE at 1, 6, and 12 months. Systemic embolism was defined by both clinical and objective evidence of the sudden loss of end-organ perfusion, confirmed by laboratory tests and imaging techniques; symptomatic DVT/PE was defined in any patients with symptoms (such as pain and/or swelling in the limbs, shortness of breath, chest pain) and laboratory tests (i.e., d-dimer elevation) suggestive for these conditions and objective documentation of DVT or PE assessed by imaging techniques (vascular and organ echography, Doppler ultrasound, intravenous contrast-enhanced computed tomography). Secondary safety endpoints were the number of bleeding events, assessed according to the Bleeding Academic Research Consortium (BARC) scale and Thrombolysis in Myocardial Infarction (TIMI) definitions at 1, 6, and 12 months. The secondary efficacy endpoint was edoxaban plasma concentrations at steady state. The main features of the ORIGAMI study design are summarized in Figure 1.

**TABLE 1:** Inclusion and exclusion criteria.

Inclusion criteria	Exclusion criteria
PEG	Contraindications to edoxaban
Indication for long-term oral anticoagulation	Less than 18 years of age
Ability to express informed consent	Less than 30-day life expectancy
	Inability to express informed consent

PEG, percutaneous endoscopic gastrostomy.

## Results

A total of 12 patients were enrolled. They were predominantly males (58%) with a mean age of 73 (±5). One patient had a previous history of heart failure with reduced ejection fraction and three patients (25%) had a history of ischemic heart disease, two with previous PCI (17%), and one with previous CABG (8%). A total of four patients (33.3%) were already taking a DOAC, whilst no patients were previously on VKA therapy. The main underlying condition leading to PEG was ALS (10/12); one patient suffered from Steinert myotonic dystrophy and one from sequelae of an ischemic stroke. Edoxaban 60 mg was administered in one patient, while a reduced dose of 30 mg was administered in 11 patients, due to reduced eGFR (5/12) or low body weight (6/12). A detailed description of patients’ characteristics has been reported previously (11) and in Table 2.

**TABLE 2:** Patients’ baseline characteristics.

Characteristics	Overall (*n* = 12)
Age (mean years ± SD)	73 ± 5
Female sex [*n*/total *n* (%)]	5/12 (42)
BMI (mean Kg/m^2^ ± SD)	23 ± 3
Systolic blood pressure (mean mmHg ± SD)	112 ± 12
Diastolic blood pressure (mean mmHg ± SD)	66 ± 8
Heart rate (mean ± SD)	74 **±** 12
**Medical history [*n*/total *n* (%)]**	
HFrEF	1/12 (8)
HFpEF	0/12 (0)
Prior PCI	2/12 (17)
Prior CABG	1/12 (8)
**Risk factors**	
Hypertension [*n*/total *n* (%)]	7/12 (58)
Diabetes mellitus [*n*/total *n* (%)]	1/12 (8)
Dyslipidaemia [*n*/total *n* (%)]	3/12 (25)
Smoker [*n*/total *n* (%)]	0/12 (0)
CHADS-VASc (mean ± SD)	3 ± 1
HAS-BLED (mean ± SD)	2 ± 1
**Echocardiography at enrollment [mean ± SD (*n*)]**	
LVEF (%)	59 ± 10 (5)
E/A	0.9 ± 0.6 (3)
E/E’	8.2 ± 2.0 (4)
**Full blood count and coagulation at baseline (mean ± SD)**	
Haemoglobin (g/dl)	11.1 ± 2.4
Red blood cell count (×10^^12^/l)	3.8 ± 0.8
White blood cell count (×10^^9^/l)	7.4 ± 3.1
Neutrophils (%)	65.7 ± 9.7
Lymphocytes (%)	25.1 ± 9.5
Monocytes (%)	6.5 ± 2.0
Platelet count (×10^^9^/l)	300 ± 133
aPPT (second)	38 ± 4
INR	1.1 ± 0.1
Fibrinogen (mg/dl)	424 ± 115
**Blood chemistry at baseline (mean ± SD)**	
Glucose (mg/dl)	114 ± 31
Total cholesterol (mg/dl)	147 ± 39
HDL (mg/dl)	43 ± 14
LDL (mg/dl)	88 ± 35
TGL (mg/dl)	133 ± 104
ALT (Ul/l)	17 ± 8
AST (Ul/l)	16 ± 4
LDH (Ul/l)	146 ± 34
Albumin (g/l)	31 ± 5
Cystatin C (mg/l)	1.8 ± 0.6
**Pharmacotherapy at baseline [*n*/total *n* (%)]**	
Previous VKA	0/12 (0)
Previous DOAC	4/12 (33)
ASA	1/12 (8)
P_2_Y_12_ inhibitor	1/12 (8)
Statin	3/12 (25)
Beta-blocker	7/12 (58)
Ca2^+^ channel blocker	1/12 (8)
Diuretic	9/12 (75)
Insulin	1/12 (8)
ACEi/ARB	1/12 (8)
**Condition leading to the PEG procedure (*n*/total *n*)**	
ALS	10/12
Steinert myotonic dystrophy	1/12
Ischemic stroke	1/12
**Edoxaban dose (*n*/total *n*)**	
Edoxaban 60 mg	1/12
Edoxaban 30 mg due to eGFR 15–50 mL/min/1.73 m^2^	5/12
Edoxaban 30 mg due to body weight < 60 Kg	6/12

BMI, body mass index; ALS, amyotrophic lateral sclerosis; AF, atrial fibrillation; CAD, coronary artery disease; HFrEF, heart failure with reduced ejection fraction; HFpEF, heart failure with preserved ejection fraction; PCI, percutaneous coronary intervention; CABG, coronary artery bypass graft; LVEDV, left ventricular end diastolic volume; LVESV, left ventricular end systolic volume; LVEF, left ventricular ejection fraction; PT, prothrombin time; INR, international normalized ratio; HDL, high density lipoprotein; LDL, low density lipoprotein; TGL, triglycerides; ALT, alanine transaminase; AST, aspartate transaminase; LDH, lactate dehydrogenase; ASA, acetyl salicylic acid; ACEi, angiotensin-converting-enzyme inhibitors; ARB, angiotensin II receptor blockers.

The primary composite endpoint of ischemic stroke, systemic embolism, or symptomatic DVT/PE did not occur in any patients at 12 months follow-up.

**FIGURE 2:**
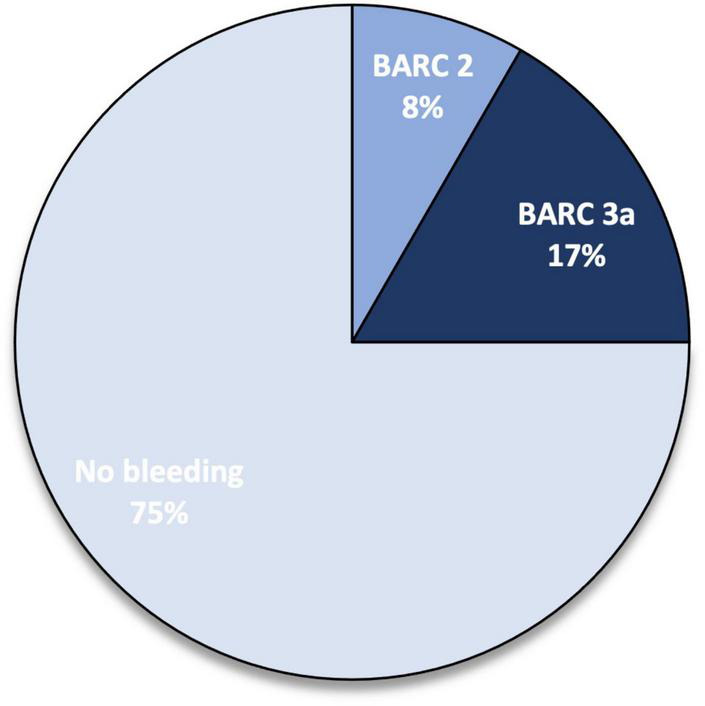
Percentage of bleeding events at follow-up. BARC, Bleeding Academic Research Consortium.

**FIGURE 3:**
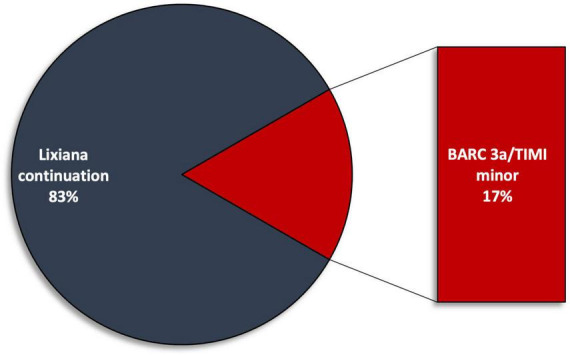
Edoxaban withdraw rate due to bleeding events during follow up. BARC, Bleeding Academic Research Consortium; TIMI, Thrombolysis in Myocardial Infarction.

Three (25%) minor bleeding occurred during follow-up: one episode of haematuria at 1-month follow-up (TIMI minor, BARC 3a), one episode of rectorrhagia at 6-month follow-up (TIMI minimal, BARC 2), and one episode of haematuria at 12-month follow up (TIMI minor, BARC 3a) (Figure 2). These events led to edoxaban withdrawal in two patients, starting parenteral anticoagulation with low molecular weight heparin (at a therapeutic dose adjusted according to body weight and renal function) (Figure 3). No major bleedings were recorded. Steady-state edoxaban plasma levels were at the therapeutic range in all patients, in agreement with recent findings (12), considering the threshold of 25 ng/ml, as recommended by kit producer and in line with data reported by the International Council for Standardization in Haematology (ICSH) Recommendations for Laboratory Measurement of Direct Oral Anticoagulants (13) and its recent update (14), despite evidence about a precise therapeutic range for edoxaban are lacking (15). The mean plasma concentration was 208.5 (± 78.6) ng/ml.

**FIGURE 4:**
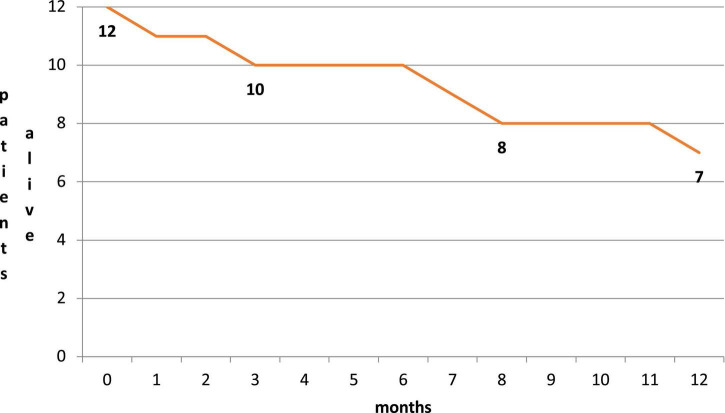
Survival curve during follow up.

Five patients died during follow-up: one died at a 1-month follow-up due to acute respiratory failure, and one died at a 6-month follow-up due to acute respiratory failure. Three patients died during the 12-month follow-up period due to acute respiratory failure, sepsis, and intestinal occlusion (Figure 4), respectively. All five deaths were related to non-cardiovascular causes and were in line with the natural history of the pre-existing severe disease. During follow-up, none of the caregivers reported limitations in the assumption of the compound related to the route of administration, which was deemed feasible and preferred to parenteral administration.

## Discussion

The longest available follow-up of the ORIGAMI pilot study confirmed our preliminary results, that edoxaban administration *via* PEG is feasible and appears safe and effective in fragile, comorbid patients, resulting in therapeutic edoxaban plasma concentrations.

Direct oral anticoagulants have a remarkable efficacy/safety ratio, and a predictable anticoagulant effect and are recommended by ESC guidelines over VKA for the prevention of stroke and systemic embolism in adult patients with indication for long-term anticoagulation (1). Despite the same guidelines reporting the possibility of administering these drugs in crushed form through a nasogastric tube, it is not mentioned whether it is safe and effective to administer DOACs through PEG. This route of administration, in fact, was not tested in the main trials that investigated the clinical effectiveness and safeness of four available DOACs (16–19). Nevertheless, the following sub-analysis and further evidence supported the use of this class of drug in clinical practice also in fragile patients and extreme conditions (3, 4, 20, 21). Our study confirmed, for the first time, that edoxaban administration through PEG is feasible and well tolerated by fragile patients.

Percutaneous endoscopic gastrostomy implantation is a relatively safe procedure, but patients are often severely comorbid (i.e., neurological disorders, cancer, stroke), with elevated frailty score indexes, and at an increased risk of complications and death. This is particularly true for patients with ALS, which was the leading cause of PEG implantation in our population (22). In such a class of patients, periprocedural and long-term anticoagulation can be challenging to be managed, and in clinical practice, parenteral anticoagulants are often preferred, leading to several issues related to patients and/or caregivers (subcutaneous injections twice a day) acceptance and guidelines adherence to the prescribed treatment. Even regular monitoring and adjustment required in case of treatment with VKAs can be arduous for these patients, that usually have poor mobility and poor quality of venous access. Since the quality of life of these patients is reduced, every effort should be made to simplify their prescriptions and to provide easier routes of administration of a specific treatment, aiming to increase the perceived effectiveness/safety ratio profile (23). Edoxaban is a direct Factor Xa inhibitor, has a linear and predictable pharmacokinetic profile and is administered once daily (24). In our study, edoxaban proved to be a possible solution for the clinically relevant issue of anticoagulation need in fragile patients with PEG, having the advantage of avoiding daily subcutaneous administrations compared to low molecular weight heparins or weekly/monthly blood sampling to monitor effectiveness compared to VKAs, while minimizing the risk of bleeding, known to worse prognosis (25), and being effective in preventing thromboembolic events. Moreover, patients and caregivers found this route of administration practical, well tolerated and with a net clinical benefit on quality of life.

Lastly, it is worth noting that, to date, a specific therapeutic range has not been identified for edoxaban plasma concentration, and the drug plasma levels at steady state ranged quite a lot in previous studies (15, 26, 27). We found as well a large variety of concentrations among patients of our study, ranging from 51.0 to 344.0 ng/ml, but in line with those expected after therapeutic doses as suggested by the ICSH (14).

As previously discussed, we recognize that this study presents several limitations (11). First, it was a non-randomized, single-arm study. However, to assess the feasibility and preliminary data about effectiveness and safety, the study design was deemed appropriate. Second, the small sample size prevents any definitive conclusion about clinical outcomes. This sample size was chosen based on the pilot nature of the study, and our results will be useful for the rationale of future investigations (28). Our findings, moreover, are in line with those of a previous study in 30 healthy adults that support the use of edoxaban tablets crushed and administered *via* a nasogastric tube (29). A prospective, larger, randomized trial is therefore needed. Lastly, we observed a high mortality rate (5 out of 12) of included patients. Nevertheless, these were all non-cardiovascular deaths related to the severe diseases from which patients already suffered, and in line with their natural history (30).

## Conclusion

In conclusion, we first described and confirmed that edoxaban is apparently safe and effective if administered *via* PEG in clinical practice. This route of assumption results in a therapeutic concentration of the drug being well tolerated by fragile patients, providing a remarkable improvement in the patient’s and caregivers’ quality of life and adherence to the therapy.

## Data availability statement

The raw data supporting the conclusions of this article will be made available by the authors, without undue reservation.

## Ethics statement

The studies involving human participants were reviewed and approved by the Fondazione Policlinico Universitario Agostino Gemelli IRCCS. The patients/participants provided their written informed consent to participate in this study.

## Author contributions

LC, RL, and DD’A conducted the study. RD and MS supported the realization of the study. LC and RL had a leading role in writing the manuscript. MG and FC had a leading role in manuscript revision. FC, GC, AR, GP, and AA had a supporting role in manuscript revision. All authors contributed to the article and approved the submitted version.
